# Top-Down Cognitive and Linguistic Influences on the Suppression of Spontaneous Otoacoustic Emissions

**DOI:** 10.3389/fnins.2018.00378

**Published:** 2018-06-08

**Authors:** Viorica Marian, Tuan Q. Lam, Sayuri Hayakawa, Sumitrajit Dhar

**Affiliations:** ^1^Department of Communication Sciences and Disorders, Northwestern University, Evanston, IL, United States; ^2^Department of Psychological Sciences, Loyola University, New Orleans, LA, United States

**Keywords:** otoacoustic emissions, individual differences, speech perception, bilingualism, cognitive control, executive function

## Abstract

Auditory sensation is often thought of as a bottom-up process, yet the brain exerts top-down control to affect how and what we hear. We report the discovery that the magnitude of top-down influence varies across individuals as a result of differences in linguistic background and executive function. Participants were 32 normal-hearing individuals (23 female) varying in language background (11 English monolinguals, 10 Korean-English late bilinguals, and 11 Korean-English early bilinguals), as well as cognitive abilities (working memory, cognitive control). To assess efferent control over inner ear function, participants were presented with speech-sounds (e.g., /ba/, /pa/) in one ear while spontaneous otoacoustic emissions (SOAEs) were measured in the contralateral ear. SOAEs are associated with the amplification of sound in the cochlea, and can be used as an index of top-down efferent activity. Individuals with bilingual experience and those with better cognitive control experienced larger reductions in the amplitude of SOAEs in response to speech stimuli, likely as a result of greater efferent suppression of amplification in the cochlea. This suppression may aid in the critical task of speech perception by minimizing the disruptive effects of noise. In contrast, individuals with better working memory exert less control over the cochlea, possibly due to a greater capacity to process complex stimuli at later stages. These findings demonstrate that even peripheral mechanics of auditory perception are shaped by top-down cognitive and linguistic influences.

## Introduction

Understanding language and speech comes naturally to most people. This fluency and apparent ease belies the intricate processes that contribute to the comprehension of speech, starting with our ability to perceive sounds and extending to the extraction of meaning. Complex cognitive tasks like the disambiguation of words based on context are affected by individual differences in executive functions like working memory (Gunter et al., 2003). Additionally, central cognitive processes may step in to compensate for deficits in peripheral processing, as when there is damage to inner ear structures (e.g., the decline comprehension hypothesis; Wong et al., 2009, 2010). This idea aligns with findings that successful speech perception is associated with greater working memory capacity and cognitive reserve (Rudner and Lunner, 2014). Here, we propose that general cognitive processes may not only compensate for, but may directly influence peripheral structures. We examine whether differences in cognitive abilities, such as in inhibitory control and working memory, affect how well the brain can exert top-down control over the mechanics of the inner ear (Khalfa et al., 2001; Perrot et al., 2006; see Terreros and Delano, 2015 for a review of top-down, corticofugal modulation of activity in the auditory periphery).

In addition to cognitive abilities, we explore whether bilingual experience, which has been linked to greater cognitive control, can enhance top-down influences on auditory processing. There is reason to believe this could be the case, as experiences other than with language have been shown to influence lower levels of sensory processing. For example, musical experience can improve speech perception through top-down influences on subcortical brainstem activity (Parbery-Clark et al., 2009a,b, 2011) and peripheral structures like the cochlea (Perrot et al., 1999; Brashears et al., 2003; Strait et al., 2012; see Perrot and Collet, 2014 for review). Strait and Kraus (2011) suggest that these effects of musical experience may be partly driven by enhanced cognitive control, yet little has been done to directly examine the relationship between cognitive abilities and inner ear activity. Given the critical, yet difficult task of extracting specific signals like speech amid noise, it follows that attention, working memory, and other cognitive abilities could exert influence at multiple levels of processing, beginning with one of the earliest steps—provision of amplification by the outer hair cells (OHCs) of the cochlea.

The OHCs are responsible for amplifying auditory inputs in a precise frequency-specific manner before they are passed on to the brain. This process of amplification can be modulated by the cholinergic auditory efferent system via myelinated fibers of the medial olivocochlear bundle projecting from the superior olivary complex to the OHCs (Cooper and Guinan, 2006). This auditory efferent network allows signals from the brain to travel down to modulate OHC amplification. The precise functions of this top-down influence are still under investigation, but some have suggested that it may aid in signal or speech detection under adverse acoustic conditions by improving the signal-to-noise ratio in the output of the cochlea (Kawase et al., 1993; Kumar and Vanaja, 2004). If cognitive control is associated with how well individuals can exert top-down influence over peripheral processes, the benefits of cognitive control on speech perception may be partially due to activities that occur much earlier in the processing stream than initially supposed. To test this idea, we utilize established techniques (Talmadge et al., 1993; Guinan, 2006; Deeter et al., 2009; Zhao and Dhar, 2011, 2012) to measure sounds emitted by the ear, called Spontaneous Otoacoustic Emissions (SOAEs).

SOAEs are associated with the amplification function of OHCs (Guinan, 2006) and can be detected non-invasively by a miniature microphone placed in the sealed ear canal. Presenting sounds to the contralateral ear can excite the medial olivocochlear efferent system, leading to a reduction of amplification in the cochlea, and a subsequent suppression of sounds created by amplification (i.e., SOAEs; Mott et al., 1989). Because the cochlear amplifier is non-linear by nature, gain is highest when the level of input is the lowest. Thus, by monitoring change in SOAE levels, we potentially gather information about changes in cochlear amplification while the cochlear amplifier is operating at maximal gain. Additionally, by examining SOAEs in response to contralateral stimulation, we are able to avoid the contamination that would result from presenting sounds directly to the test ear, as well as from external stimuli that would be needed to elicit emissions using other types of OAEs (e.g., DPOAEs, TPOAEs). It should be noted that the amount of SOAE suppression can vary considerably across individuals (Harrison and Burns, 1993), and can be influenced by processes other than efferent control over the cochlear amplifier. For instance, increasing or decreasing ear canal air pressure has been found to influence both the frequency and amplitude of SOAEs, likely due to changes in impedance of the middle ear (Schloth and Zwicker, 1983; Naeve et al., 1992). Examination of efferent modulation of SOAE level and frequency has typically involved controls to rule out any involvement of either pressure change or a muscular reflex in the outer and middle ears (e.g., Zhao and Dhar, 2010). In this report we do not actively alter pressure in the ear canal and use stimulation levels well below those known to activate the middle ear muscle reflex. We therefore regard the changes in SOAE level and frequency observed here as a useful indicator of efferent activity, as has been commonly done in the literature (Mott et al., 1989; Harrison and Burns, 1993).

We measured SOAEs during a speech perception task while collecting measures of cognitive abilities to examine whether there is a reliable relationship between cognitive control and modulation of the inner ear's amplifier. As noted, a potential function of the auditory efferent network is to flexibly improve the signal-to-noise ratio to enhance speech perception. Individuals with greater cognitive control may therefore exhibit greater alteration of cochlear amplification and SOAE amplitude, in response to stimuli.

Lastly, if enhanced cognitive control can influence the mechanics of inner ear functions, we may generate other hypotheses for the types of factors that could also impact sound perception, such as bilingual experience. This is based on research demonstrating enhanced cognitive abilities in bilinguals due to the unique challenges of managing multiple languages. Bilinguals access words from both languages, even when only one is relevant (Marian and Spivey, 2003; Thierry and Wu, 2007; Bialystok et al., 2008). This requires inhibition of the non-target language, and extensive experience juggling multiple linguistic systems can enhance cognitive control (Bialystok, 2001; Bialystok et al., 2004; Costa et al., 2008; Blumenfeld and Marian, 2011). Language coactivation can also cause one language to interfere with the other (Rodriguez-Fornells et al., 2005), potentially leading to deficits in processing speech under noisy conditions (Mayo et al., 1997; Bradlow and Bent, 2002). Increased difficulty in identifying speech may also result from less practice with the sounds and words of a particular language, either because it was acquired later in life or because time was split between multiple languages (Gollan et al., 2008). Whatever the cause of the bilingual deficit in speech perception, this increased difficulty may make modulation of cochlear gain especially important in order to help improve the signal-to-noise ratio and facilitate comprehension. If we were to find an effect of bilingualism on SOAEs, it would suggest that consequences of language experience could extend beyond higher-level cognition to impact the very functioning of the auditory sensory organ. To examine this possibility, we compared the amplitude of SOAEs collected during a speech perception task in English-speaking monolinguals and Korean-English bilinguals. The bilinguals varied in their ages of English acquisition, allowing us to evaluate whether the effect of bilingualism on efferent activity is moderated by the magnitude of bilingual experience.

## Materials and methods

### Ethical approval

This study was carried out in accordance with the recommendations of Northwestern University's Institutional Review Board. The protocol (STU00000295) was approved by Northwestern University's Institutional Review Board. All subjects gave written informed consent in accordance with the Declaration of Helsinki.

### Participants

Fifty-one adults participated in this study. Of the initially recruited participants, eight males and eleven females were excluded because they did not have SOAEs in baseline measurements. The remaining 32 participants (23 female) were between the ages of 18–26 and had normal hearing as assessed by both self-report and the presence of SOAEs, which is a strong indicator of hearing function (McFadden and Mishra, 1993). Table **2** in the Results section displays demographic information collected using the LEAP-Questionnaire (Marian et al., 2007) for the monolingual and bilingual participants.

### Materials and procedure

SOAEs were measured in one ear while syllables such as /ba/ and /ga/ were presented to the other ear. The participant's task was to identify the sound by clicking on one of six possible syllables which were visually presented in a circular array (“ba,” “ga,” “da,” “ta,” “pa,” and “ka”). We compared the change in SOAE amplitudes across participants with varying cognitive abilities as measured by the NIH cognitive toolbox (Gershon et al., 2013) to assess whether there was a relationship between cognitive abilities and auditory processing. These included a flanker task to measure inhibitory control and attention, the Dimensional Change Card Sort task to assess mental flexibility, a speeded pattern matching task to assess processing speed, as well as tests to assess working memory and receptive vocabulary. As many aspects of cognition are correlated, we conducted a factor analysis to reduce the number of variables for our model using the “psych” (Revelle, 2017) and “GPArotation” (Bernaards and Jennrich, 2005) packages in R, utilizing an oblimin rotation and the minimum residual (OLS) technique. We label the first factor as “Working Memory,” since the working memory measure had a positive loading of 0.999 and no other factors had loadings greater than 0.3. We label the second factor as “Control,” since it had the most positive loading for the Flanker task (0.922), followed by Pattern Comparison (0.525), and Card Sort (0.509). Complete factor loadings can be found in Table 1. Composite scores were created for each of these factors by summing the Z-standardized task scores which had been multiplied by their factor loadings.

**Table 1:** Factor loadings for NIH toolbox measures.

	**“Control”**	**“Working memory”**
Vocabulary		0.223
Flanker	0.922	
Working memory		0.999
Card Sort	0.509	−0.119
Pattern	0.525	−0.128

Stimuli consisted of speech syllables lasting ~500 ms, produced by a female native speaker of English. There were six syllables presented: /ba/, /da/, /ga/, /pa/, /ta/, and /ka/. Auditory sound pressure level (SPL) was normalized at 60 dB peak to peak.

Before beginning the experiment, we evaluated probe position and microphone sensitivity by measuring the sound pressure in response to a sweeping frequency tone ranging from 200 Hz to 20 kHz over a 10 s period, played through an RME FireFace 400 soundcard at a sampling rate of 44.1 kHz. The sound pressure recorded in the ear canal was normalized to a pre-recorded spectrum obtained in a reflection free (long) tube. The frequency position of resonance peaks in this normalized output was indicative of the depth of insertion of the probe, the peak levels and low frequency trajectory indicated goodness of probe fit. Next, participants sat in silence for 3 min while baseline recordings were obtained from both ears. A fast Fourier transform (FFT) was then conducted on these recordings to generate the frequency spectrum to identify SOAEs in each ear. SOAE recordings during the speech perception task were obtained from the ear that showed the highest number of SOAEs between the 1,000 and 10,000 Hz range. Sound was presented to the contralateral ear through an ear bud with disposable inserts.

After baseline SOAEs were obtained, participants began the speech perception task. Trials began with the presentation of either a video or static image. The speaker in the video remained motionless for the first 1,500 ms. After 1,500 ms, the speech sound was played and the video/image remained onscreen for an additional 500 ms after the speech sound finished. When the video/image was removed, participants were presented with a six-item forced-choice display from which participants had to click on the sound they heard with a mouse. After indicating their response, participants began the next trial.

There was a total of 240 trials that were split into ten blocks. After every block, participants were given a short break of ~2 min. At the halfway point of the experiment, participants were given a longer break (5–10 min) and given an opportunity to move around. The duration of the sound perception task was ~1 h. After completing all ten blocks of the perception task, participants completed the NIH cognitive toolbox battery (Gershon et al., 2013).

### Apparatus

Videos were displayed using a 27-inch iMac computer running MATLAB 2010. The screen resolution on the computer was set at 2,560 × 1,440 pixels. Sound was delivered unilaterally to a participant's ear through an ear bud. The medial olivocochlear response was measured using the contralateral SOAE suppression procedure. The SOAEs were recorded using an ER-10B+ microphone and preamplifier (20-dB gain) attached to a probe while sound was presented using an ER-2 speaker. Both were fitted with an ER10-14 disposable foam ear tip (Etymotic Research, Elk Grove Village, Illinois, USA). All recordings were conducted in a sound treated room.

### Data analysis

We conducted a 22,050-point short time Fourier transform on all SOAE recordings with a Hamming window size of 16,384 points and a hop size of 4,096 points. SOAEs with highly variable levels (*SD* > 6 dB) across four 30-s periods in the baseline were removed. We filtered out SOAEs whose peaks were within 5 Hz of a multiple of 60 Hz as these may have been generated or influenced by line noise. For each remaining SOAE, we extracted the local peak within 25 Hz of the baseline SOAE for each sample, and then took the average of the peaks over 500-ms beginning at the onset of the syllable. We also computed the average peak level over a 500-ms period before trial onset to use as the baseline for the trial. Analyses were conducted on the difference in peak levels between the speech period and the baseline period for each trial. Although efferent-induced changes in SOAE level can manifest over longer periods of time, the majority of the observed change has been shown to occur on a fast time scale over 10 s of ms. Thus, by measuring across a 500-ms window, we expected to detect the majority of the total efferent-induced change. Intensity differences were computed in dB. Initial analyses of language experience and cognitive abilities were conducted using two separate linear models in the R environment (R Core Team, 2016). The presence of a video vs. a static image did not interact with either of the variables of interest and therefore were not factored into the final analysis. For both language experience and cognitive abilities, the differences in peak levels of individual SOAEs were entered as the dependent variable, and Language Group (for language experience) or the composite z-scores for Control, Working Memory, and the interaction (for cognitive abilities) were entered as predictors. Subsequently, the residuals from the cognitive ability model were entered as the dependent variable with language group as the predictor to determine the effect of language experience controlling for cognitive abilities. To determine the effect of language experience on the Control and Working Memory measures, each of the cognitive ability measures were separately entered as the dependent variable with language group as the predictor.

## Results

### Effect of cognitive abilities on SOAEs

**Figure 1:**
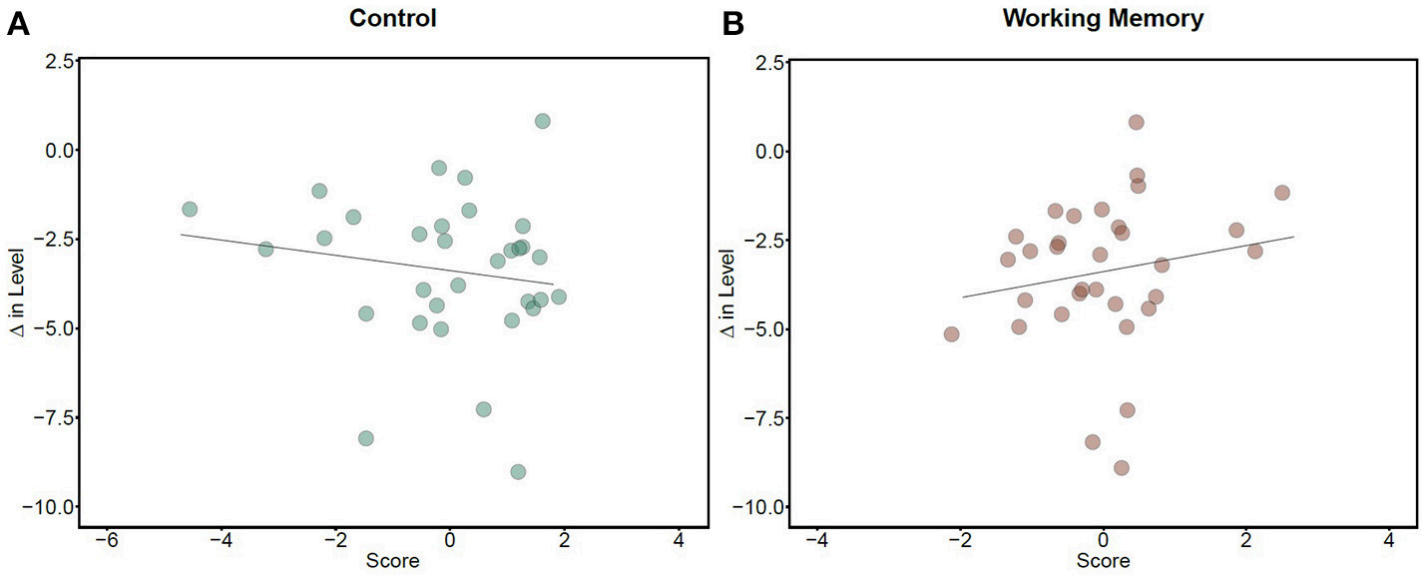
Changes in SOAE levels and cognitive abilities. Dots represent individual subjects' (*N* = 32) average change in SOAE level (dB). More negative values for SOAE change represent greater suppression in response to stimuli compared to the 500 ms baseline period preceding it. More positive values for cognitive measures indicate better performance. **(A)** shows that greater cognitive control was associated with more suppression of SOAE levels during speech processing (*r* = −0.16), while **(B)** shows that greater working memory was associated with less suppression (*r* = 0.18).

Analyses revealed a main effect of Control, where higher scores were associated with larger changes in SOAE amplitude (β = −0.10, SE = 0.03, *t* = −3.81, *p* < 0.001; Figure 1A). This pattern suggests that individuals scoring higher on tasks requiring inhibitory control, mental flexibility, and processing speed showed greater inhibition of cochlear gain in response to stimuli. Contrary to the effect of Control, however, we find that greater Working Memory is associated with *smaller* changes in SOAE intensity (β = 0.32, SE = 0.04, *t* = 7.32, *p* < 0.001; Figure 1B). The interaction between the two measures was significant (β = −0.09, SE = 0.02, *t* = −3.94, *p* < 0.001). The two cognitive ability measures thus have opposite effects on auditory efferent activity, with greater suppression of SOAEs for individuals with more control, but lower working memory capacity.

### Effect of bilingual experience on cognitive abilities

Bilinguals scored significantly higher than monolinguals on the Control measure (β = 1.37, SE = 0.54, *t* = 2.55, *p* = 0.016; see Table 2). This is consistent with past research demonstrating a bilingual advantage for tasks that require inhibitory control (Bialystok, 2001; Bialystok et al., 2004; Costa et al., 2008; Blumenfeld and Marian, 2011). For Working Memory, on the other hand, monolinguals outperformed bilinguals (β = −0.72, SE = 0.35, *t* = −2.05, *p* = 0.049). The two measures were not correlated with each other [*r* = −0.196, *t*_(30)_ = −1.09, *p* = 0.281], and no effect of age of English acquisition was found among bilinguals for either the Control (β = −0.07, SE = 0.07, *t* = −0.96, *p* = 0.349) or Working Memory measures (β = −0.002, SE = 0.06, *t* = −0.04, *p* = 0.968). The lower working memory scores in bilinguals were unexpected, as past research has generally shown either no differences between monolinguals and bilinguals (e.g., Bialystok et al., 2008; Engel de Abreu, 2011), or a bilingual advantage (e.g., Blom et al., 2014). A possible explanation is that these lower scores are driven by differences in English proficiency rather than working memory. This would be in line with Calvo et al.'s (2016) proposal that bilinguals may experience some difficulty with verbal working memory tasks as a result of challenges associated with language comprehension rather than working memory *per se*. The working memory task utilized in this study required recalling items in English, and the bilinguals reported lower levels of English proficiency compared to the monolinguals. This explanation is supported by a significant positive relationship between Working Memory and self-reported English proficiency (β = 0.44, SE = 0.20, *t* = 2.21, *p* = 0.036). No relationship was observed between English proficiency and the Control measure (β = 0.15, SE = 0.34, *t* = 0.44, *p* = 0.66). Table 2 displays average scores and demographic information for monolinguals and bilinguals, with bilinguals divided into those who acquired English before the age of 7 (early) and after 7 (late).

**Table 2:** Cognitive measures and demographic information.

	**Monolinguals (*N* = 11)**	**Late bilinguals (*N* = 10)**	**Early bilinguals (*N* = 11)**	**Mono vs. late**	**Mono vs. early**	**Late vs. early**
Control	−0.90 (1.96)	0.36 (1.19)	0.57 (1.06)	ns	0.044	ns
Working memory	0.48 (1.07)	−0.54 (0.85)	0.01 (0.87)	0.026	ns	ns
Age	21.18 (1.60)	21.3 (2.36)	20.91 (2.54)	ns	ns	ns
Female (*N*)	8	8	7	ns	ns	ns
English proficiency	9.79 (0.40)	8.59 (0.98)	9.36 (0.75)	0.006	ns	ns
Korean proficiency	–	9.0 (0.97)	8.06 (2.19)	–	–	ns
English AoA	0.18 (0.40)	9.11 (1.45)	3.0 (1.89)	<0.0001	0.0001	<0.0001
Korean AoA	–	0.22 (0.44)	1.36 (1.36)	–	–	0.022

*The table displays averages with standard deviations in parentheses. Self-rated proficiency was rated on a scale from 0 to 10. AoA, or age of acquisition, indicates the age (in years) at which participants reported beginning to learn each language. The last three columns display significant p-values of t-tests comparing each group to each other*.

### Effect of bilingual experience on SOAEs

Monolinguals and bilinguals did not differ from each other in the number of tested SOAE frequencies per subject (*M* = 2.09 and 2.14, respectively; *p* = 0.859), the average tested SOAE frequency (*M* = 2291.77 and 2056.67 Hz; *p* = 0.423), accuracy in the speech perception task (*M* = 67.8% and 66.3%; *p* = 0.322), or the distribution of test ears (63.6% and 47.6% right ears; *p* = 0.472). Monolinguals did, however, have lower baseline SOAE levels than bilinguals (*M* = −4.91 and −2.92 dB SPL; β = 1.95, SE = 0.11, *t* = 16.96, *p* < 0.001). See Table 3 for more detailed SOAE characteristics.

**Table 3:** SOAE characteristics for monolinguals and bilinguals.

	**Monolinguals (*N* = 11)**	**Bilinguals (*N* = 21)**
*N* Peaks	23	45
Average peaks per subject	2.09	2.14
Frequencies (Hz)	1,068–6,444	1,132–4,538
Percent right ears	63.6%	47.6%
Baseline level in right ear (dB SPL)	−5.13	−3.41
Baseline level in left ear (dB SPL)	−4.52	−2.48
Stimulus level in right ear (dB SPL)	−7.53	−6.65
Stimulus level in left ear (dB SPL)	−8.45	−6.42

**Figure 2:**
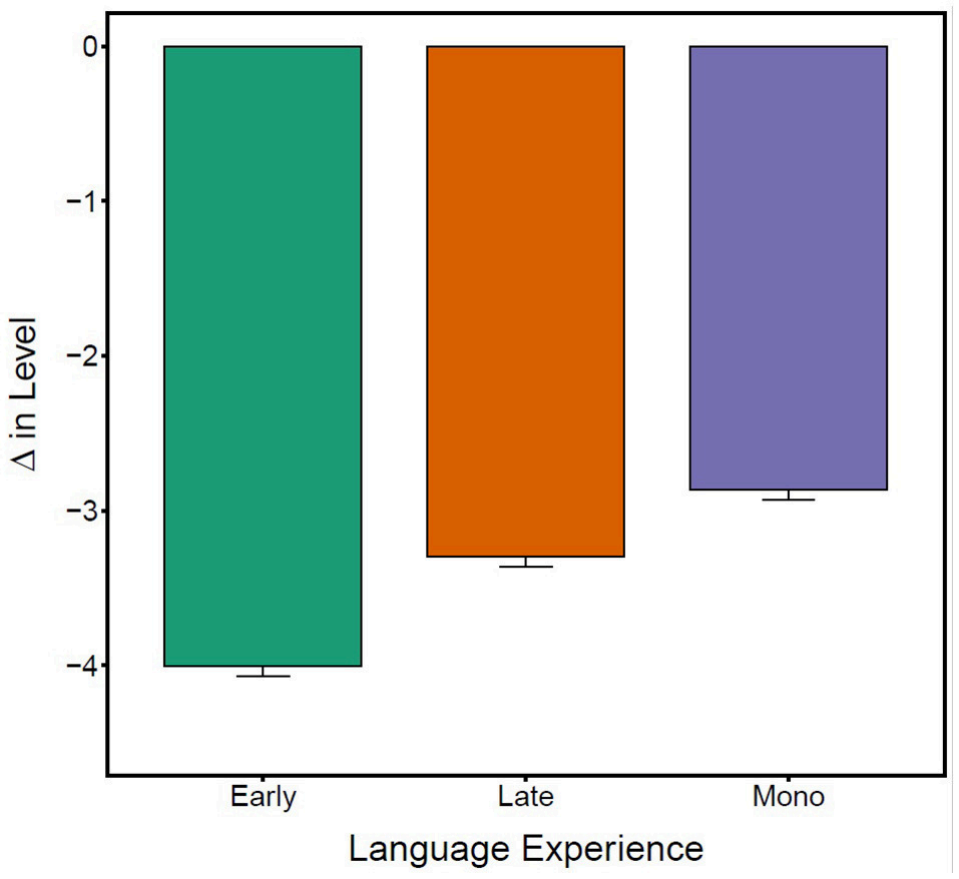
Average change in SOAE level (dB) for early bilinguals (*N* = 11), late bilinguals (*N* = 10), and monolinguals (*N* = 11). More negative values indicate greater suppression to stimulus relative to baseline. Error bars represent standard errors.

Controlling for baseline SOAE levels, greater changes in SOAE levels were detected among bilinguals relative to monolinguals (β = −0.83, SE = 0.08, *t* = −10.40, *p* < 0.001). In bilinguals, greater SOAE suppression was associated with earlier English acquisition (β = 0.06, SE = 0.01, *t* = 4.34, *p* < 0.001). To illustrate the bilingual pattern, Figure 2 divides the participants with bilingual experience into early (age of English acquisition <7 years old) and late bilinguals (>7).

To observe the effect of language background on SOAEs after controlling for cognitive ability, the residuals from the cognitive ability model were entered as the outcome variable with language group entered as the predictor. We found that the effect of bilingual experience remains, with bilinguals showing greater inhibition of SOAEs relative to monolinguals (β = −0.31, SE = 0.08, *t* = −3.92, *p* < 0.001). In other words, being bilingual leads to greater inhibition of cochlear gain even after accounting for differences in cognitive ability. All reported results remain significant when including test ear (left or right) as a covariate in the statistical models (all *p* < 0.001).

## Discussion

Early peripheral functions of auditory perception are linked to individual differences in both cognitive abilities and language experience. Higher cognitive control scores (i.e., better inhibition, mental flexibility, and processing speed) were correlated with greater suppression of SOAEs, likely reflecting increased inhibition of cochlear gain. Top-down control over the amplification of sound may enhance the perception of transient signals like speech by essentially turning down the volume of background noise and thereby improving the signal-to-noise ratio (Kawase et al., 1993; Kumar and Vanaja, 2004). Individuals with greater inhibition, mental flexibility, and processing speed may exert better control through the efferent system and have an advantage for speech perception. This would be consistent with work demonstrating that general cognitive abilities can enhance the perception of speech (Rudner and Lunner, 2014). The present research differs from prior work, however, by demonstrating that cognitive control can affect speech perception at a much lower level than generally presumed, by altering the mechanics of the auditory sensory organ. To our knowledge, this is the first evidence in support of individual differences in cognitive abilities leading to differences in sensory processing at the auditory periphery.

In contrast to abilities captured by the “Control” factor, *lower* working memory scores led to greater reductions in SOAE level between stimulus presentations and the baseline periods preceding them. One interpretation of this result is that individuals with less capacity for manipulating complex stimuli in working memory rely on early filtering to a greater extent in order to more efficiently allocate limited resources. This notion is consistent with past work demonstrating that greater cognitive load leads to a reduction in the magnitude of auditory-evoked brainstem responses, indicative of an early gating mechanism (Sörqvist et al., 2012). It also aligns with Lavie and Tsal's (1994) proposal that the stage at which attentional selection occurs varies as a function of both perceptual load (i.e., the complexity of the sensory stimuli) and cognitive capacity. When perceptual load exceeds an individual's cognitive capacity, early selection mechanisms will be employed to filter noise and distractors. However, if sufficient attentional resources remain, even irrelevant stimuli will be processed. Individuals with sufficient working memory capacity to manage irrelevant stimuli may exert less control at the level of the cochlea.

We also found that bilinguals exhibited greater SOAE suppression in response to stimuli compared to monolinguals. This effect is consistent with prior work suggesting that bilingualism enhances cognitive control (Bialystok, 2001; Bialystok et al., 2004; Costa et al., 2008; Blumenfeld and Marian, 2011). It is additionally consistent with the fact that musical experience leads to greater suppression of cochlear gain (Perrot et al., 1999; Brashears et al., 2003; Strait et al., 2012), given that musicianship and bilingualism have been shown to elicit similar cognitive benefits (Schroeder et al., 2015). What was not fully expected was that the effect of bilingualism on efferent control was independent of the cognitive abilities measured in the present study. Bilinguals may suppress cochlear gain more than monolinguals to compensate for their greater difficulty in comprehending speech in noise (Mayo et al., 1997; Bradlow and Bent, 2002). In other words, bilinguals may exert more efferent control over cochlear function, possibly not because they possess superior cognitive control, but because they utilize their available capacity to a greater extent to make up for deficits in speech perception. A lifetime of dealing with complex linguistic environments may develop habits of processing that have consequences for both cognition and perception.

One potential limitation of the current study is that English was not the dominant language for all bilingual participants. As a result, the observed bilingualism effect could conceivably be a “non-native” effect (Weiss and Dempsey, 2008). We do not believe this explanation is likely, however, as the bilingualism effect was even more pronounced for the higher proficiency, early bilinguals than for the lower proficiency, late bilinguals. As previously noted, a second potential limitation is that SOAEs are an indirect measure of efferent activity, and could therefore be influenced by other factors such as the amount of air pressure in the ear canal (Schloth and Zwicker, 1983). While it is possible that the cognitive and linguistic effects observed in the present study are the result of changes to structures other than the cochlea, efferent modulation is a more likely explanation as, to our knowledge, there is no reason or evidence to suggest that either cognitive abilities or language background would influence ear canal air pressure.

In conclusion, the present results illustrate that our ability to hear does not result from a passive, feed-forward unidirectional process, but rather an elaborate network of activity that involves both bottom-up and top-down influences. Our auditory system responds flexibly to the environment at multiple levels of processing and functions differently across situations as well as people. Here, we demonstrate that individual differences in both cognitive abilities and language experience have consequences for auditory processing that extend beyond the brain to the earliest, most peripheral structures.

## Data availability

The datasets generated and/or analyzed during the current study are available from the corresponding author on request.

## Author contributions

VM, SD, and TL designed the study. TL collected the data. TL and SH analyzed the data and drafted the manuscript. VM, SD, and SH edited and finalized the manuscript. All authors contributed to interpretation of the results.

### Conflict of interest statement

The authors declare that the research was conducted in the absence of any commercial or financial relationships that could be construed as a potential conflict of interest.
